# Job strain in public transport drivers: Data to assess the relationship between demand-control model indicators, traffic accidents and sanctions

**DOI:** 10.1016/j.dib.2018.05.036

**Published:** 2018-05-18

**Authors:** Sergio Useche, Luis Montoro, Boris Cendales, Viviola Gómez

**Affiliations:** a University of Valencia, Valencia, Spain; b El Bosque University, Bogotá, Colombia; c University of Los Andes, Bogotá, Colombia

**Keywords:** JDC, Job Demand-Control Model, JCQ, Job Content Questionnaire, Public transport drivers, Professional driving, Work stress, Job strain, Demand-Control Model, Working conditions, Traffic accidents, Traffic fines

## Abstract

This Data in Brief (DiB) article examines the association between the Job Demand-Control (JDC) model of stress and traffic safety outcomes (accidents and sanctions) in public transport drivers (*n* = 780). The data was collected using a structured self-administrable questionnaire composed of measurements of work stress (Job Content Questionnaire), and demographics (professional driving experience, hours and days working/driving per week). The data contains 4 parts: descriptive statistics, bivariate correlations between the study variables, analysis of variance (ANOVA) and Post-Hoc comparisons between drivers classified different quadrants of the JDC model. For further information, it is convenient to read the full article entitled “*Working conditions, job strain and traffic safety among three groups of public transport drivers*”, published in Safety and Health at Work (SHAW) [1] (Useche et al., 2018).

**Specifications Table**

**Table:** 

Subject area	*Psychology*
More specific subject area	*Occupational psychology, work stress, risk management, road safety in the field of public transportation.*
Type of data	*Tables, graph, database*
How data was acquired	*Original data collection*
Data format	*Filtered and analyzed*
Data source location	*Bogotá, Colombia*
Data accessibility	*Presented data is derived from the original database reported in the article. It also contains the full database obtained for the study, as supplementary material.*

**Value of the data**

• This data provides information on the psychosocial working conditions, driving experience and hourly intensity, traffic fines and accidents experienced by Colombian public transport drivers.
• The work-stress (job strain) data can be compared with other groups of professional drivers assessed through the JDC model.
• The data could be analyzed according to the type of service (i.e. vehicle driven) of professional drivers working in the field of public transportation.
• The data can be used by other researchers and road safety practitioners to analyze the psychosocial working conditions of public transport drivers.

## 1 Design, materials and methods^1^

### 1.1 Participants

In this cross-sectional study, participants were a sample of *n =* 780 male professional drivers working in public transport companies of Bogotá (Colombia): 448 (57.4%) city bus drivers, 195 (17.6%) taxi drivers, and 137 (25%) inter-urban bus drivers, with a mean age of *x̄ =* 41.13 [18–76 range] (*SD =* 11.3), and an average driving experience of *x̄ =* 17.6 (*SD =* 9.87) years. Their average driving intensity was *x̄ =* 72.58 (*SD =* 9.15) hours per week. For this study, women were excluded from crossed analyses due to their underrepresentation in the public transport drivers’ occupational group (approximately 98.5% of the total sample was composed of males).

### 1.2 Questionnaire

The Karasek's Job Content Questionnaire (JCQ) [2], a self-report tool for the assessment of psychosocial factors at work widely used in different occupational groups [3] -including professional drivers [4]-, was used for this study.

In its validated version for Colombian workers [5], the JCQ is composed of 27 items grouped in six scales: support from supervisors (4 items, α = 0.87; example item: *My supervisor or boss helps the work to be done*), peer support (4 items, α = 0.79 example item: *My colleagues help the work to be done*), skill discretion (6 items, α = 0.75 example item: *My job requires that I learn new things*), decision authority (3 items, α = 0.69; example item: *My job allows me to make a lot of decisions on my own*), psychological demands (6 items, α = 0.66; example item: *My job requires working very fast*), and job insecurity (4 items, α = 0.53; example item: *The stability in my job is good*) [4], [6]. Decision latitude or “control at work” was calculated as the sum of use of skills and decision making, and job strain as the ratio between psychological demands and decision latitude (demands/decision latitude). Additionally, the participants completed a brief demographic questionnaire which asked for their age, driving experience, type of vehicle/service operated, work schedules (driving hours per week, week days driving and weekend days driving), road crashes (accidents) and penalties (fines) registered in the last two years.

### 1.3 Statistical analysis

First of all, basic descriptive analyses (i.e. means and frequencies related to the study´s variables) were obtained, and bivariate correlations were used to examine the association between some key working conditions, and psychosocial work factors in road safety outcomes (traffic accidents and sanctions in a period of two years). The “job strain score” provided by the JCQ was complemented with the quadrant-based approach, which classifies the workers above the sample median for demands and below the median for decision latitude in the “job strain” or “high strain quadrant”, below median for demands and above the median for decision latitude in the “low strain quadrant”, below the medians for demands and decision latitude in the “ passive job quadrant”, and above the medians for demands and decision latitude in the “active job quadrant”. Finally, a comparative test (Post-Hoc) was performed with the aim of comparing accident and sanctions reported by drivers based on their JDC-quadrant (i.e. passive work, low-strain, high strain and active job).

## 2 Data

**Fig. 1:**
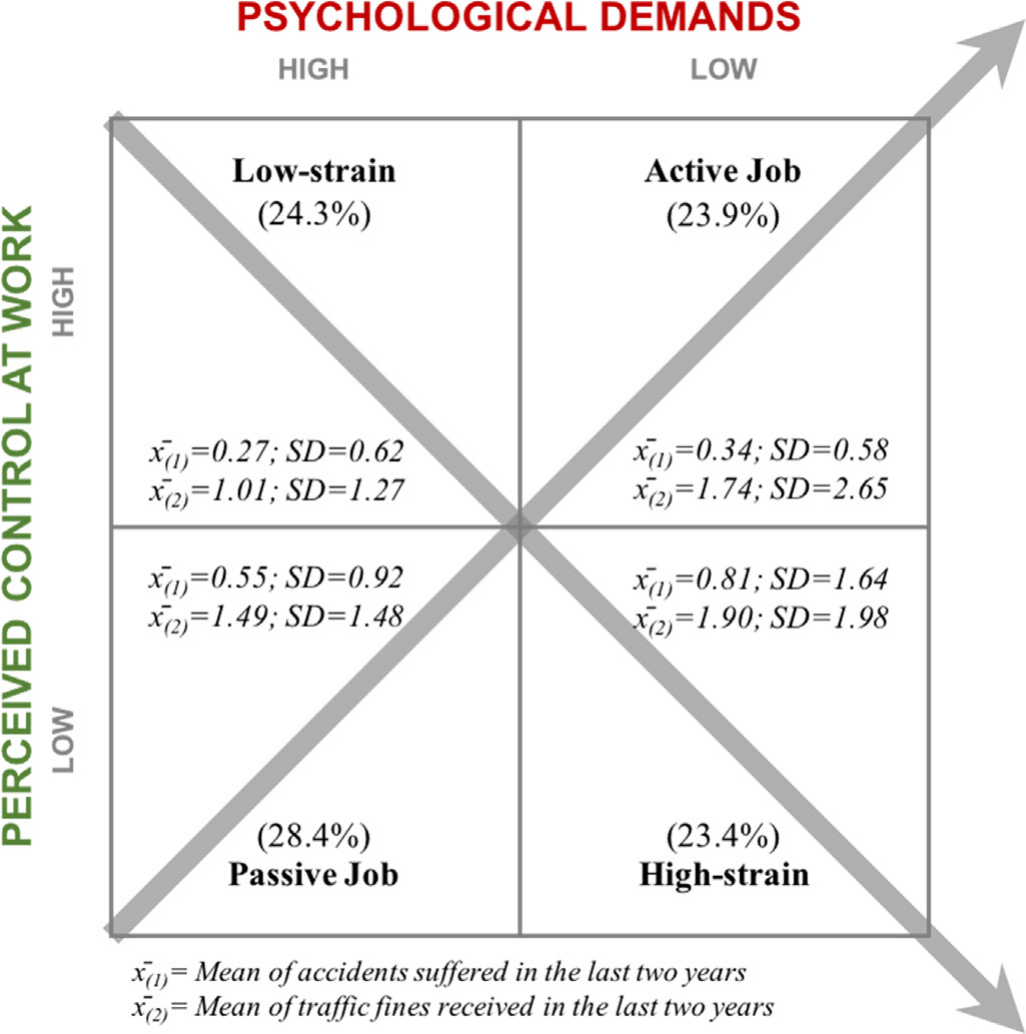
JCQ's quadrant-based distribution for levels of perceived control at work and psychological demands.

The dataset of this article provides information on the entire set of psychosocial work factors typically addressed by the JDC model (i.e. social support, control, psychological demands, job insecurity and the job strain index). Table 1 shows the descriptive statistics obtained for all JCQ subscales, and Table 2 shows the partial correlations between study factors, controlling for perceived social support. Fig. 1 categorizes professional drivers according to the “quadrant approach” of the JDC model, and shows their specific mean rates of accidents and traffic fines. Finally, Table 3 summarizes the results of a Post-Hoc (Tukey-HSD) analysis, which examines the mean differences in traffic accidents and fines between public transport drivers in different quadrants of the Demand-Control model.

**Table 1:** Descriptive statistics of the variables contained in the data set.

**Variable**	**N**	**Minimum**	**Maximum**	**Mean**	**Std. Error**	**Std. Dev.**
Age	769	18	76	41.13	.402	11.14

*Driving experience and habits*						
Driving Experience (years)	769	1	52	18.38	.356	9.87
Weekdays Driving	761	1	5	4.94	.012	.33
Driving Hours per Week	752	15	77	72.58	.334	9.15
*Job Content Questionnaire*						
Supervisor Support	750	4	16	11.58	.121	3.32
Peer (Co-Worker) Support	748	4	16	11.28	.107	2.92
General Social Support	744	8	32	22.87	.199	5.44
Use of Skills	756	14	48	36.80	.191	5.26
Decision Making	760	12	48	39.25	.305	8.40
Control at Work	755	26	96	76.05	.445	12.22
Psychological Demands	752	12	48	32.36	.271	7.42
Job Insecurity	732	4	15	6.78	.085	2.30

**Table 2:** Partial correlations between study variables *.

**Variable**		**Statistic**	**2**	**3**	**4**	**5**	**6**	**7**	**8**
**1**	Age	Correlation	−.018	.805	−.118	.019	−.131	−.122	−.167
		Sig. (2-tailed)	.637	.000	.002	.630	.001	.002	.000
**2**	Hours Driven per Week	Correlation	1	.033	.064	.181	.213	−.009	.062
		Sig. (2-tailed)	.	.402	.101	.000	.000	.827	.116
**3**	Driving Experience (years)	Correlation		1	−.057	.036	−.084	−.120	−.152
		Sig. (2-tailed)		.	.144	.352	.032	.002	.000
**4**	Job Insecurity	Correlation			1	−.092	.281	.152	.170
		Sig. (2-tailed)			.	.019	.000	.000	.000
**5**	Control at Work	Correlation				1	.099	−.168	−.067
		Sig. (2-tailed)				.	.011	.000	.086
**6**	Psychological Demands	Correlation					1	.087	.209
		Sig. (2-tailed)					.	.025	.000
**7**	Traffic Accidents (2 years)	Correlation						1	.210
		Sig. (2-tailed)						.	.000
**8**	Traffic Fines (2 years)	Correlation							1
		Sig. (2-tailed)							.

**Table 3:** Post-Hoc (Tukey HSD) analysis - Mean comparisons for traffic accidents and fines. Factor: JCQ quadrant.

**Dependent variable**	**(I) Quadrant (JCQ)**	**(J) Quadrant (JCQ)**	**Mean Diff. (I-J)**	**Std. Error**	**Sig. (p-value)**	**95% [CI]**	
						**Lower**	**Upper**
**Traffic Accidents (2 years)**	Job strain	Active Job	.471*	.111	<.001	.19	.76
		Low Strain	.542*	.110	<.001	.26	.82
		Passive Job	.263	.107	.067	−.01	.54
	Active job	Job Strain	−.471*	.111	<.001	−.76	−.19
		Low Strain	.071	.109	.915	−.21	.35
		Passive Job	−.209	.106	.199	−.48	.06
	Low strain	Job Strain	−.542*	.110	<.001	−.82	−.26
		Active Job	−.071	.109	.915	−.35	.21
		Passive Job	−.280*	.104	.038	−.55	−.01
	Passive job	Job Strain	−.263	.107	.067	−.54	.01
		Active Job	.209	.106	.199	−.06	.48
		Low Strain	.280*	.104	.038	.01	.55
**Traffic Fines (2 years)**	Job Strain	Active Job	.162	.205	.859	−.37	.69
		Low Strain	.894*	.204	<.001	.37	1.42
		Passive Job	.414	.197	.155	−.09	.92
	Active Job	Job Strain	−.162	.205	.859	−.69	.37
		Low Strain	.732*	.202	.002	.21	1.25
		Passive Job	.252	.196	.571	−.25	.76
	Low Strain	Job Strain	−.894*	.204	<.001	-1.42	−.37
		Active Job	−.732*	.202	.002	-1.25	−.21
		Passive Job	−.480	.195	.066	−.98	.02
	Passive Job	Job Strain	−.414	.197	.155	−.92	.09
		Active Job	−.252	.196	.571	−.76	.25
		Low Strain	.480	.195	.066	−.02	.98

* The mean difference is significant at the 0.05 level.

In addition, the supplementary SPSS dataset (.sav) will allow researchers to perform additional tests and comparisons using the entire set of measured variables.

## Footnotes

1 For further information, please refer to Useche et al. [1].
