# Supplementary material for: Job strain in public transport drivers: Data to assess the relationship between demand-control model indicators, traffic accidents and sanctions
Source: Data Brief. 2018 May 18;19:293–8. doi: 10.1016/j.dib.2018.05.036 (PMC5993012; doi:10.1016/j.dib.2018.05.036)
Supplement: Supplementary file 1 — Supplementary material [file mmc1.docx]

Conflict of interest

The authors declare not competing interests on this manuscript.
